# Nanocrystalline Akaganeite as Adsorbent for Surfactant Removal from Aqueous Solutions

**DOI:** 10.3390/ma6010184

**Published:** 2013-01-10

**Authors:** George Z. Kyzas, Efrosyni N. Peleka, Eleni A. Deliyanni

**Affiliations:** 1 Department of Petroleum and Natural Gas Technology, Technological Educational Institute of Kavala, Kavala 65404, Greece; 2 Department of Chemistry, Aristotle University of Thessaloniki, Thessaloniki 54124, Greece; E-Mails: peleka@chem.auth.gr (E.N.P.); lenadj@chem.auth.gr (E.A.D.)

**Keywords:** akaganeite, removal, adsorption, surfactant

## Abstract

The present study presents the effective use of nanocrystalline akaganeite for the adsorption of an anionic (SDS), a cationic (CTAB), and a nonionic (tween80) surfactant from wastewater. Equilibrium experiments, as well as thermodynamic analysis, were performed. The maximum SDS adsorption occurs at the lowest pH value (5), the opposite is observed for CTAB (pH = 11), while for tween80, the change of pH value did not affect the adsorption. The equilibrium data could be described by Freundlich and Langmuir isotherms. The maximum adsorption capacity at 25 °C (pH = 8) was 823.96 mg/g for SDS, 1007.93 mg/g for CTAB, and 699.03 mg/g for tween80. The thermodynamic parameters revealed the exothermic and spontaneity nature of the process. Also, FTIR measurements established that surfactants are adsorbed on the surface of akaganeite, replacing adsorbed water.

## 1. Introduction

Surfactants are amphiphilic compounds that contain a hydrophobic portion, which effectively repels water molecules, and a hydrophilic portion that attracts the water molecules. The use of surfactant is gradually increasing day by day. The consumption of surfactants for both industrial and domestic purpose has resulted in a worldwide production of approximately 17 million tons in 2000 (including soap), with expected future growth rates of 3%–4% per year globally, and of 1.5%–2.0% in the EU [1]. In domestic wastewater produced in households, surfactants invariably exist in a significant amount due to the enormous use of detergents for washing purposes. Surfactants have also been widely used in textiles, fibers, food, paints, polymers, cosmetics, pharmaceuticals, mining, oil recovery, pulp and paper industries, *etc.* The textile industries alone consume roughly 10% of the world surfactant production, and the wastewater of these industries (e.g., from cotton desizing and other textile finishing process) contains a high concentration of impurities, particularly surfactants [2,3,4,5]. As a consequence of their widespread use and strong resistance to biodegradation, surfactants may persist in wastewater treatment systems at relatively high concentrations [6,7].

Surfactants are harmful to human beings, fish, and vegetation. Besides the toxic effects of surfactants, their existence in waters—even below toxic levels—causes many adverse effects on biological life. They cause pathological, physiological, and biochemical effects on aquatic animals. In aquatic plant species, they have effects such as break-up of the chlorophyll–protein complex, death of the cell by damaging the membrane, in addition to delay in metabolism and growth [8]. From this perspective, surfactants should be removed from selected stages of the industrial process or wastewater emission systems. The removal of these materials from industrial wastewaters is one of the major environmental problems because of the difficulty of treating such water by conventional treatment methods.

Among various processes adopted for surfactant removal, adsorption is an effective separating process that is widely used in the water treatment field. The separating agent for adsorption is adsorbent and, consequently, the performance of any adsorptive separation or purification process is determined by the effectiveness of the adsorbent material used. While activated carbon is the most common adsorbent used for the purpose [9], many other adsorbents, among them layered double hydroxides [10], polymeric resins [11], perlite [12], alumina [2], charcoal [13], sand [14], MCM [15], or chitosan [16], have also been implemented for surfactant removal.

An important requirement for efficient removal, which is desirable by all researchers, is the high capacity of the adsorbent. Akaganeite, an iron oxyhydroxide (β-FeOOH), which was prepared in the laboratory, proved to be an excellent adsorbent for removing both anions (arsenate [17], phosphate [18], chromate [19]) and cations (cadmium [20], zinc [21]), presenting higher maximum adsorption capacities compared to other adsorbents. Also, akaganeite presented significant performance in fixed-bed experiments [22].

From a structural point of view, akaganeite (β-FeOOH) has a tetragonal structure consisting of double chains of edge-shared octahedral, which share corners with adjacent chains to form channels running parallel to the *c*-axis [23]. A certain number of extra framework anions, such as halide ions, are necessary in the structure to balance the extra protonation of oxides in the iron octahedra since akaganeite is usually synthesized in acidic solutions. The crystal structure of β-FeOOH was shown by Mackay [24,25] to be related to the hollandite structure with chloride anions in the channels, identical to the mineral akaganeite.

Since the removal of surfactants by iron oxides have not been studied in the literature yet, the overall objective of the current paper is to examine the adsorption ability of akaganeite for an anionic (sodium dodecyl sulfate, SDS), a cationic (cetyl trimethyl ammonium bromide, CTAB) and a nonionic (tween80) surfactant. Moreover, the adsorption efficiency of akaganeite for surfactants needs to be examined, since it produces new hybrid surfactant modified materials that can be used for the removal of pentavalent [17,26] and trivalent arsenic [27,28], zinc and cadmium cations [29], phosphate anions [18] from water or wastewater, with much higher adsorption capacity as compared to pure akaganeite and to other adsorbents found in the literature.

## 2. Experimental Section

Akaganeite (abbreviated hereafter as Ak) was prepared by precipitation from an aqueous solution of FeCl_3_ (0.506 mol/L with respect to Fe^3+^). For the hydrolysis process, an aqueous solution of ammonium carbonate (0.23 g/L) was used. The chloride ions were removed through a cellulose membrane by osmosis. The obtained material was freeze-dried in a bench-scale instrument. In brief, the resulting material presented the following physical characteristics: specific surface area 330 m^2^/g, crystallite size 3–6 nm, pore volume 0.35 cm^3^/g and predominant pore diameters 2.5 and 3.6 nm. The synthesis of the iron-based adsorbent was analytically described earlier [30].

For the equilibrium experiments, a constant volume of 200 mL surfactant solution was mixed with a fixed mass of adsorbent into conical flasks, in various initial concentrations. Working solutions were prepared fresh daily for each batch test and the pH of the solution was adjusted when necessary using 0.1 mol/L HNO_3_ and/or 0.1 mol/L NaOH solution and was determined with a Crison 2000 pH-meter. The flasks were put in a shaker bath at room temperature for 24 h, at the maximum shaking rate 200 rpm, to allow the adsorption of surfactants, until the solution reached equilibrium. This contact time allows the dispersion of adsorbent and surfactants to reach equilibrium conditions, as found by preliminary experiments. Following sorption, the suspension was filtered by using a membrane filter of 0.45 μm and the filtrates were analyzed for surfactants concentration. Analysis of surfactants remaining in the solution was determined spectrophotometrically, following the standard method.

Electrophoretic mobility measurements of akaganeite particles, both in the presence and absence of surfactants, were performed in a microelectrophoresis apparatus (Rank Brothers MK II). The velocities of at least 20 particles in each direction of the electric field were measured at the two stationary layers of the capillary tube. The measurements were made at constant room temperature. The pH of the suspensions was varied by adding small amounts of HNO_3_ or NaOH.

For the estimation of the concentration of anionic, as well as the cationic surfactants, the volumetric method that includes the two-phase titration with two indicators was adopted. In the case of anionic surfactant, the titration proceeded with cationic surfactant standard solution, while for the estimation of the cationic surfactant, the titration proceeded with anionic surfactant standard solution. The indicators used consisted of two substances of contrasting colors, which made the overall color change more distinctive. The mixed indicators were a mixture 1:1 (w/w) of the anionic disulfine blue VN 150 (4',4''-diaminodiethyltriphenylmethane-2,4 disulfonate) and the cationic dimidium bromide (2',7-diamino-9-phenyl-10-methylphenanthridinium bromide). At the commencement, the organic phase is pink and the aqueous phase is blue due to dissolved dimidium bromide anionic surfactant salt and disulfine blue, respectively. At the end point, the organic phase turns to gray for the titration of the anionic surfactant, while the opposite is observed for the titration of the cationic surfactant, where the organic phase turns pink from blue [31,32]. For the estimation of tween80 (nonionic surfactant), the volumetric method with the Dragendorff reagent was adopted. The titration proceeded with EDTA solution until the yellow color (owing to bithiourea) disappears [33].

Fourier transform infrared (FTIR) spectroscopy was performed from 4000 to 300 cm^−1^ with a Perkin–Elmer Spectrum 2000 spectrophotometer. Samples of the adsorbent before and after surfactant adsorption were ground with special grade KBr in a fixed ratio, in an agate mortar. The same amount of mixed powder was also used to prepare the pellet for FTIR. All measurements were carried out at room temperature.

## 3. Results and Discussion

From a first view of the surfactant’s chemistry, surfactants are generally classified and listed in Table 1, depending on the nature of the hydrophilic group [34].

**Table 1:** Different types of hydrophilic groups of surfactant molecules and their main application.

Class	Head group	Main applications
Anionic	–COO^−^Na	Soaps
	–SO_3_^−^Na	Synthetic detergent
	–OSO_3_^−^Na	Detergents, personal care products
	–OPO_3_^−^Na	Corrosion inhibitors, emulsifiers
	–(OCH_2_CH_2_) *_n_*OSO_3_^−^Na	Liquid detergents, toiletries, emulsifiers
Cationic	–N(CH_3_)_3_^+^Cl^−^	Bitumen emulsions
	>N(CH_3_)_2_^+^Cl^−^	Fabric and hair conditioners
Zwitterionic	–N^+^(CH_3_)_2_CH_2_COO^+^	Shampoos, cosmetics
	–N^+^(CH_3_)_2_CH_2_SO_3_^+^	
Nonionic	–(OCH_2_CH_2_) *_n_*OH	Detergents, emulsifiers

By plotting solid phase concentration against liquid phase concentration, it is possible to depict the equilibrium adsorption isotherm. Various isotherm equations have been used to describe the equilibrium characteristics of adsorption. Both the adsorption mechanism and the surface properties and affinity of the adsorbent can be understood from the equation parameters and the underlying thermodynamic assumptions of these isotherm models. In this study, Langmuir (Equation (1)) [35] and Freundlich (Equation (2)) [36] and isotherms have been applied:

(1) $${\text{Q}}_{e}=\frac{{\text{Q}}_{max}{\text{K}}_{L}{\text{C}}_{e}}{1+{\text{K}}_{L}{\text{C}}_{e}}$$

(2) $${\text{Q}}_{e}={\text{K}}_{F}{\text{C}}_{e}^{1/\text{n}}$$

where Q_e_ is the quantity of solute (surfactant) adsorbed per unit weight of solid adsorbent (Ak), C_e_ is the concentration of solute in the solution at equilibrium, K_F_ and 1/n are constants indicating the adsorption capacity and the adsorption intensity respectively (1/n < 1) and K_L_ is an energy term, which varies as a function of surface coverage strictly due to variations in the heat of adsorption and Q_max_ is the maximum loading capacity.

The experimental value of Q_e_ (mg/g) was calculated using the mass balance equation:

(3) $${\text{Q}}_{e}=\frac{({\text{C}}_{0}-{\text{C}}_{e})\text{V}}{\text{m}}$$

where m is the mass of adsorbent; V is the volume of adsorbate; C_0_ is the initial concentration of solute in the solution.

**Figure 1:**
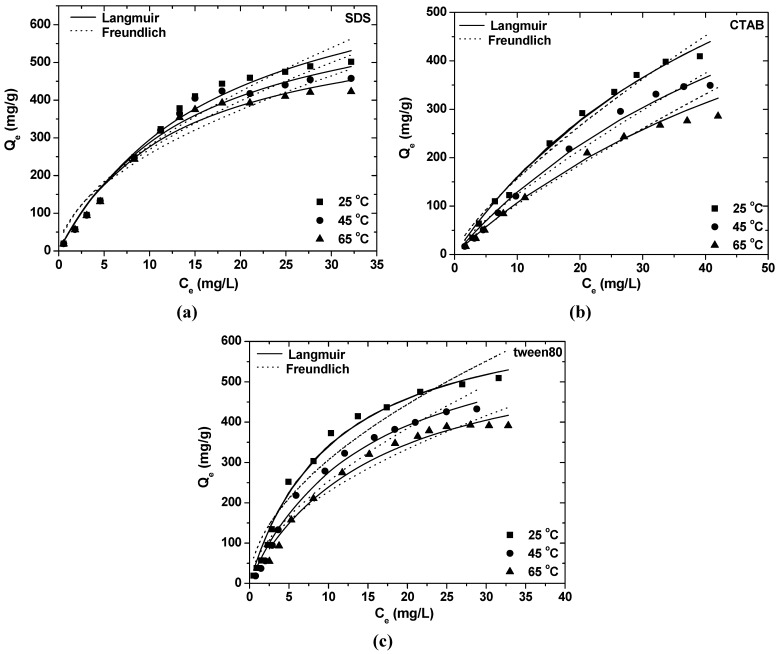
Adsorption isotherms of (**a**) sodium dodecyl sulphate (SDS); (**b**) cetyl trimethyl ammonium bromide (CTAB) and (**c**) tween80 at three different temperatures (conditions: [Ak] = 0.5 g/L; pH = 8.0; t = 24 h).

The molecular structure of the surfactant influences the shape of the isotherm in various ways. Within a homologous series, it is found that increasing length of the hydrocarbon chain generally increases the magnitude of adsorption at the plateau and diminishes with increasing size of the hydrophilic head group on the hydrophobic solid [37]. The equilibrium results of SDS, CTAB and tween80 on akaganeite are presented in Figure 1, respectively.

The amphoteric nature of akaganeite, makes it a suitable adsorbent material for anions, cations and nonionic species. Each data series was fitted by employing non-linear regression analysis. The estimated values of parameters of the two models, as well as the correlation coefficients, are listed in Table 2.

According to these coefficients, both models could adequately predict experimental data. The characteristic feature in these figures is the decrease of adsorption loading as the temperature of the bulk raises, indicating that the adsorption of surfactants is an exothermic process.

Regarding Langmuir model the adsorption capacity Q_max_ was found to decrease from 823.96 to 631.06 mg SDS per g Ak, from 1007.93 to 867.44 mg CTAB per gram Ak and from 699.03 to 613.01 mg tween80 per gram Ak, as the temperature was increased. Regarding the Freundlich model and the adsorption of SDS on Ak, K_F_ values were increased with increasing temperature, showing that the rate of adsorption was increased. In case of CTAB and tween80, K_F_ values were decreased with increasing temperature, showing that the rate of adsorption was decreased. The latter contradiction was used to show the superiority and better fit of the Langmuir model for the present study.

**Table 2:** Equilibrium parameters for adsorption of surfactants on akaganeite.

Surfactant	θ (°C)	Langmuir constants			Freundlich constants		
		R^2^ (–)	Q_max_ (mg/g)	K_L_ (L/mg)	R^2^ (–)	K_F_ (mg^1−(1/n)^ L^1/n^ g^−1^)	n (–)
SDS	25	0.983	823.96	0.056	0.950	70.74	1.67
	45	0.976	710.49	0.069	0.933	74.31	1.78
	65	0.975	631.06	0.079	0.925	75.45	1.87
CTAB	25	0.992	1007.93	0.019	0.981	27.25	1.32
	45	0.992	935.21	0.016	0.983	19.93	1.26
	65	0.996	867.44	0.014	0.991	15.09	1.19
tween80	25	0.989	699.03	0.096	0.945	89.86	1.87
	45	0.989	671.38	0.070	0.958	64.71	1.66
	65	0.981	613.01	0.065	0.939	63.45	1.83

In order to shed light on the above, a thermodynamic analysis was performed to the data in Figure 1. The thermodynamic equilibrium constant K_0_ for the adsorption process was determined by plotting ln(Q_e_/C_e_) *versus* Q_e_ and extrapolating to zero Q_e_ using a graphic method. Regression straight lines are fitted through the data points by the least squares method. The intersection with the vertical axis gives the value of K_0_. The change in free energy (ΔG^0^) was estimated from the following relationship:

(4) $$\text{Δ}{\text{G}}^{0}=-\text{RTln}({\text{K}}_{0})$$

The changes in enthalpy (ΔH^0^) and entropy (ΔS^0^) were obtained according to the Van’t Hoff equation [38]:

(5) $$\text{Δ}{\text{G}}^{0}=\text{Δ}{\text{H}}^{0}-\text{TΔ}{\text{S}}^{0}$$

The plot of ΔG^0^ *versus* T was found to be linear. Values of ΔS^0^ and ΔH^0^ were evaluated from the slope and intersect of the Van’t Hoff plots. Additionally, the isosteric heat of adsorption (ΔH*_x_*) was calculated by the means of the integrated form of the Clausius–Clapeyron equation given by the relation:

(6) $$\text{Δ}{\text{H}}_{x}=2.303\text{R}\frac{{\text{T}}_{1}{\text{T}}_{2}}{{\text{T}}_{2}-{\text{T}}_{1}}(\text{log}({\text{C}}_{{\text{e}}_{1}})-\text{log}({\text{C}}_{{\text{e}}_{2}}))$$

where T_1_, T_2_ are two of the constant (particular) temperatures (*i.e.*, 25 and 45 °C; 25 and 65 °C; 45 and 65 °C), and C_e1_, C_e2_ are the concentration of solute in the solution at equilibrium at the above two constant temperatures.

The estimated thermodynamic parameters for the adsorption of SDS, CTAB and tween80 on Ak are presented in Table 3.

**Table 3:** Thermodynamic parameters for adsorption of surfactants on akaganeite.

Adsorbent	θ (°C)	lnK_0_ (–)	ΔG^0^ (kJ/mol)	ΔH^0^ (kJ/mo)l	ΔS^0^ (kJ/mol K)	ΔH*_x_* (kJ/mol)
SDS	25	3.839	–9.511	–7.170	0.0079	–7.05
	45	3.681	–9.732			
	65	3.497	–9.828			
CTAB	25	2.919	–7.234	–9.564	0.0081	–9.68
	45	2.597	–6.868			
	65	2.459	–6.912			
tween80	25	4.983	–12.346	–8.048	0.0366	–7.00
	45	4.160	–10.994			
	65	3.872	–10.882			

The negative values of ΔG^0^ suggest that the process is spontaneous with the high preference of surfactants for Ak. The negative values of ΔH^0^ suggest the exothermic nature of the process. The positive values of ΔS^0^ indicated an increased randomness. The negative values of ΔH*_x_* suggest also the exothermic nature of the process.

In our previous work [22], akaganeite performed well as a fixed-bed adsorbent for anions and cations. Based on these results, the performance of this material for waste water treatment in the presence of other compounds and its regeneration is going to be examined. Preliminary tests on regeneration showed that regeneration can proceed with a low pH while in presence of surfactants onto akaganeite; anions (arsenates, phosphates) and cations (zinc, cadmium) can be adsorbed onto its surface. Thus, akaganeite cannot be claimed to be a commercially competitive material, but a good adsorbent presenting a high value of maximum adsorption capacities for a variety of water contaminants.

Comparing briefly the experimental findings to those of literature, Table 4 presents a short list of the published maximum load of various surfactant adsorbents, including the present study. Akaganeite, as compared to the other adsorbent materials, has high—in fact, the highest—adsorption capacity, which made it a useful adsorbent material for the treatment of water or wastewater containing surfactants, as well as trace metal ions.

**Figure 2:**
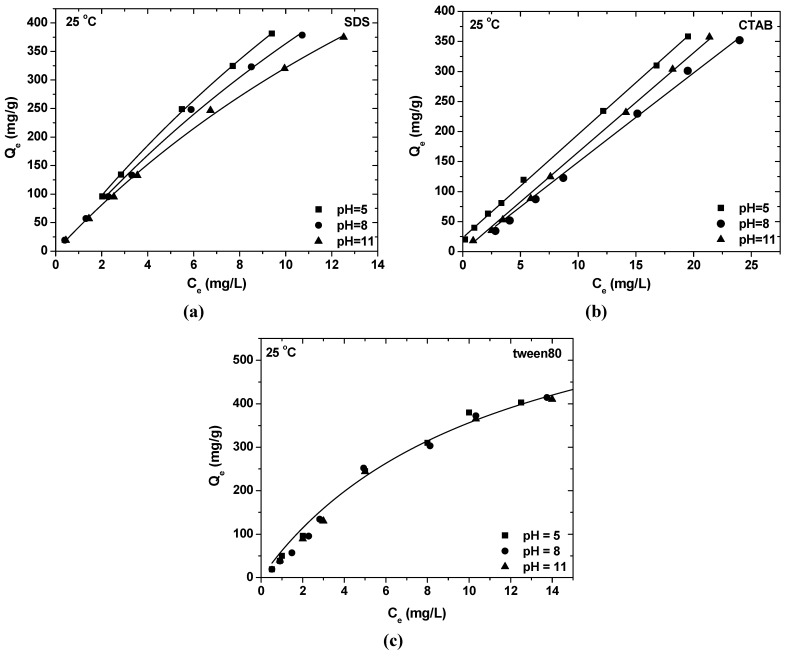
Effect of pH value on the adsorption of (**a**) SDS; (**b**) CTAB and (**c**) tween80 onto Ak (conditions: [Ak] = 0.5 g/L, t = 24 h, θ = 25 °C).

Figure 2 presents the influence of solution pH on the sorption of surfactants onto akaganeite at three different pH values (5, 8, 11). The maximum SDS adsorption occurs at the lowest pH value (Figure 2a). This is due to the fact that, in this pH range, the surface of Ak is positively charged and electrostatically attracts the negatively charged –SO_4_^2−^ surfactant headgroups. The opposite is observed in case of CTAB (Figure 2b), where the highest pH enforces the adsorption. At this pH range, the surface of akaganeite is negatively charged and electrostatically attracts the positively charged –NH_4_^+^ surfactant headgroups. In the case of nonionic tween80 (Figure 2c), the change of pH value has no reflection on surfactant sorption onto Ak due to the absence of polar groups of the surfactant.

**Table 4:** Comparison of Q_max_ for various adsorbents.

Adsorbent	Surfactant	Q_max_ (mg/g)	Q_max_ (mg/g m^2^)	Reference
akaganeite	SDS	823.96	2.50	this study
sand	SDS	1.31	–	[39]
Ca-montmorillonite	SDS	7.00	0.92	[40]
activated carbon	SDS	271	0.32	[41]
carbon	SDS	55.68	0.58	[42]
paper fiber	SDS	0.30	0.20	[42]
akaganeite	CTAB	1007.93	3.05	this study
perlite	CTAB	38.40	16.70	[43]
powdered activated carbon	CTAB	400.90	0.86	[44]
silica gel waste	CTAB	73.85	0.28	[45]
akaganeite	tween80	699.03	2.12	this study

**Figure 3:**
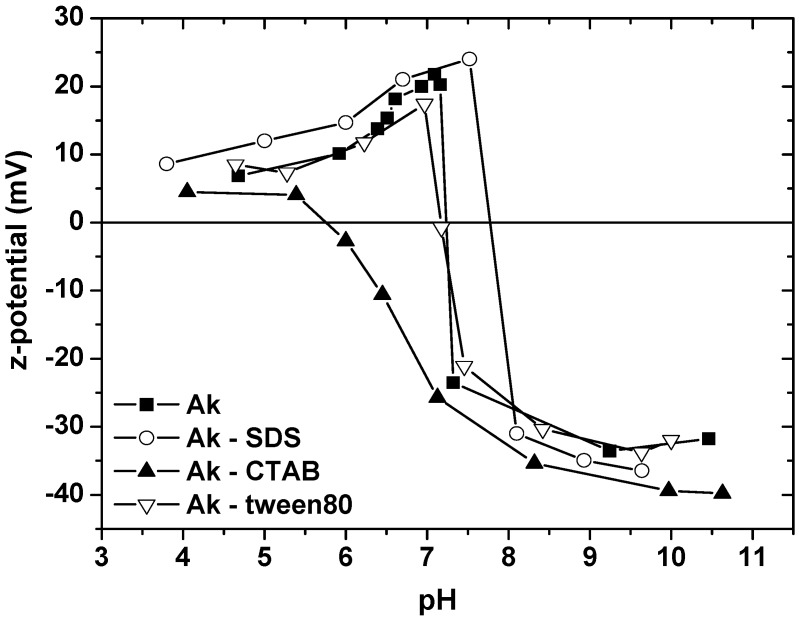
Electrokinetic measurements, expressed as zeta-potential, of the akaganeite after surfactants adsorption as a function of solution pH.

In Figure 3, the electrokinetic measurements of the system under investigation is presented. Akaganeite particles immersed in a polar liquid medium, water, acquire a surface charge. At the same time, this charge can arise in many different ways. The most important mechanism of the surface charge generation comes via ionization of surface functional groups: if a surface contains acidic functional groups, their dissociation gives rise to a negatively charged surface; conversely, a basic surface takes on a positive charge. In both cases, the magnitude of the surface charge, *i.e.*, the degree of surface ionization, depends on the acidic or basic strength of the surface functional groups and on pH of the solution. The surface charge can be reduced to zero (at the point of zero charge, PZC) by suppressing the surface ionization by decreasing pH in the former case or by increasing pH in the latter case. Metal oxides, carrying hydroxyl surface functional groups, exhibit the amphoteric behavior and both a positively and negatively charged surface can be obtained by varying pH. The electrically charged surface is physically unstable and tends to be neutralized.

The PZC for akaganeite was found to be initially 7.3, but when CTAB was adsorbed, it decreased from this value to about 5.8. The specific adsorption of CTAB species makes the surface of akaganeite more negatively charged, which results in a shift of the isoelectric point of adsorbent to a lower pH value. Therefore, specific adsorption rather than a purely electrostastic interaction is further confirmed from the drop of isoelectric point at the aqueous akaganeite/CTAB interface. On the other hand, the PZC of akaganeite after SDS addition was slightly shifted from 7.3 to 8.0 approximately and this indicates a weak chemisorption or perhaps, a combination of physical and chemical adsorption.

**Figure 4:**
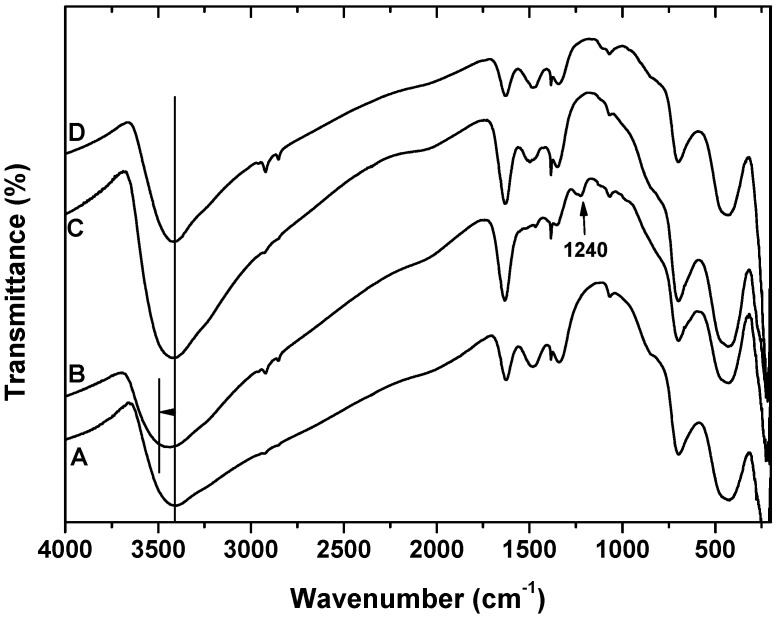
FTIR spectra of (**A**) Akaganeite (Ak); (**B**) Αk after SDS adsorption; (**C**) Ak after CTAB adsorption, and Ak after tween80 adsorption.

The infrared spectra of pure akaganeite (spectrum A), Αk after SDS adsorption (spectrum B), Ak after CTAB adsorption (spectrum C) and Ak after tween80 adsorption (spectrum D) are presented in Figure 4. The FTIR spectrum of Ak displays a broad absorption band at ~3400 cm^−1^ corresponding to the overlapping antisymmetric *ν*_3_ and symmetric *ν*_1_ (H–O–H) stretching vibrations of H-bonded water and one at the H–O–H bending region (1640 cm^−1^). Adsorbed water contributes to these regions, too. The cooperativity of hydrogen bonds like O–H...O–H...O–H may result in the band at 1064.5 cm^−1^, which corresponds to the bending vibration of hydroxyl group (M–OH) [40]. In akaganeite, there are two sets of librations, due to hydrogen bonds, *i.e.*, those of OH librational ROH 847 and 820 cm^−1^ and those of 697 cm^−1^ and 644 cm^−1^_,_ due to the two OH...Cl hydrogen bonds present. The energy translational mode of akaganeite due to the Fe–O stretches is observed at 434 cm^−1^.

In FTIR spectrum of Ak after SDS adsorption (B), the band due to the OH-stretching region of H_2_O shifts to higher frequency (3435 cm^−1^), a new band at 1240 cm^−1^ can be attributed to the stretching vibrations of the sulfate head group of the surfactant SDS (NaC_12_H_25_SO_4_) [40], while the two bands at 2921 and 2852 cm^−1^ can be attributed to the anti-symmetric and symmetric stretching vibrations of the carbon chains, respectively. These bands present small intensity in the FTIR spectra of akaganeite after CTAB adsorption. The small intensity of these adsorption bands can be attributed to the fact that the amine chains of CTAB are not highly ordered on akaganeite surface. Spectrum D, presents the spectra of akaganeite after the adsorption of the nonionic surfactant tween80. The presence of tween80 is testified by the peak of C–O–C (1098 cm^−1^), by the broadening of the band due to OH groups in the range of 3410–3320 cm^−1^, since tween80 introduces three additional –OH groups per molecule adsorbed onto the surface of akaganeite [41], and by the presence of the two bands at 2921 and 2852 cm^−1^ due to the carbon chains.

For all samples after the surfactant adsorption, the band at 1067 cm^−1^, which corresponds to the bending vibration of hydroxyl group (M–OH), was still observed, thereby showing that the surfactant has not possibly reacted with –OH functional groups of akaganeite but may be attracted electrostatically on the surface of akaganeite. The nonionic surfactant, tween80, has no net charge, but is a polar molecule that can still be attracted electrostatically to the oxide surface.

## 4. Conclusions

The present study showed that akaganeite could be effectively used for the adsorption of an anionic (SDS), a cationic (CTAB) and a nonionic (tween80) surfactant from wastewater. Equilibrium experiments, as well as thermodynamic analysis, were performed. On the basis of data obtained, the following conclusions can be deduced:

- The maximum SDS adsorption occurs at the lowest pH value, due to the fact that in this pH range, the surface of Ak is positively charged and electrostatically attracts the negatively charged –SO_4_^2−^ surfactant headgroups. The opposite is observed in the case of CTAB, where the highest pH enforces the adsorption. At this pH range, the surface of akaganeite is negatively charged and electrostatically attracts the positively charged –NH_4_^+^ surfactant headgroups. In the case of tween80, the change of pH value has no reflection on surfactant sorption onto Ak due to the absence of polar groups of the surfactant.
- Equilibrium data could be described by Freundlich and Langmuir isotherms. The maximum adsorption capacity at 25 °C and pH = 8 was found to be 823.96 mg/g for SDS, 1007.93 mg/g for CTAB and 699.03 mg/g for tween80.
- The negative values of ΔG^0^ for all surfactants suggest that the process is spontaneous with the high preference of surfactants for Ak. The negative values of ΔH^0^ for all surfactants (SDS, −7.170 kJ/mol; CTAB, −9.564 kJ/mol; tween80, −8.048 kJ/mol) suggest the exothermic nature of the process. The positive values of ΔS^0^ (SDS, 0.0079 kJ/mol; CTAB, 0.0081 kJ/mol; tween80, 0.0366 kJ/mol) indicated an increased randomness. The negative values of ΔH*_x_* (SDS, −7.05 kJ/mol; CTAB, −9.68 kJ/mol; tween80, −7.00 kJ/mol) suggest also the exothermic nature of the process.
- FTIR measurements established that surfactants are adsorbed on the surface of akaganeite, replacing adsorbed water.
